# Does Severity of Hair Loss Matter? Factors Associated with Mental Health Outcomes in Women Irradiated for Tinea Capitis in Childhood

**DOI:** 10.3390/ijerph17207388

**Published:** 2020-10-10

**Authors:** Dorit Segal-Engelchin, Shifra Shvarts

**Affiliations:** 1 Spitzer Department of Social Work, Ben-Gurion University of the Negev, Beer-Sheva 84105, Israel; 2 Faculty of Health Sciences, Ben-Gurion University of the Negev, Beer-Sheva 84105, Israel; shvarts@bgu.ac.il

**Keywords:** hair loss severity, mental health problems, irradiation treatment, women, tinea capitis

## Abstract

Hair loss resulting from childhood irradiation for tinea capitis has been linked to mental health effects in women. However, the association of hair loss severity with mental health in this population is unknown. To address this gap, this study examined the association between hair loss severity and mental health outcomes in women irradiated for tinea capitis in childhood as well as the factors that contribute to these outcomes. Medical records, held at the archives of the Israel National Center for Compensation of Scalp Ringworm Victims, were retrospectively reviewed for 2509 women who received compensation for full or partial alopecia resulting from irradiation for tinea capitis. Mental health outcomes were determined by the number of mental health conditions reported. The results show that among women with more hair loss, risk was increased for a range of mental health problems, especially social anxiety (RR 2.44, 95% CI 2.09–2.87). Hair loss severity emerged as a significant predictor of mental health, adding to the effects of other predictors such as family, social and physical health problems (β = 0.13, 95% CI 0.27, 0.56). The effects of hair loss severity on mental health outcomes were mediated by women’s negative social experiences (indirect = 0.72, 95% bias-corrected confidence interval, 0.53–1.08). Healthcare professionals supporting women with hair loss after irradiation for childhood tinea capitis should be alert to a history of severe levels of hair loss.

## 1. Introduction

Tinea capitis is a fungal infection of the scalp, primarily affecting children [1,2] and increasing in incidence in the last few decades in North America, Europe, and Africa [3,4,5]. Until the introduction of the antifungal griseofulvin in 1958, about 200,000 children worldwide [6], mainly in the United States, Europe, and the Middle East [7,8], had been treated for tinea capitis by irradiation to induce epilation. Long-term follow-up studies have demonstrated the detrimental health effects of this treatment, including meningioma, leukemia, and tumors of the brain, salivary glands, thyroid, and skin, including melanomas in the head and neck area [6,9,10,11,12,13].

Mental health issues also have been reported among patients with a history of irradiation for tinea capitis, particularly among white patients [14,15]. In comparison to patients with a history of non-irradiated tinea capitis, irradiated patients have a significantly higher incidence of diagnosed mental illness [6,14,15] and psychiatric symptoms (e.g., chronic anxiety, fears and phobias, depression, somatization, and psychotic symptoms); paranoid orientation; work problems; and maladjustment [15]. Omran et al. [15] suggested that one reason for the increased mental health vulnerability is that radiation may create stress that precipitates mental symptoms and disorders in predisposed individuals. Hair loss, which occurred in some patients irradiated for tinea capitis [6,9,11], may be a leading source of radiation-related stress, particularly among women, who tend to ascribe greater importance to their appearance than men [16,17]. 

Hair is a central feature of physical appearance [18,19], and many women view it as a central part of their identity [20,21]. Socially, hair can be viewed as an indicator of women’s femininity, sexuality, attractiveness, health, and reproductive potential [18,21,22,23]. Studies have shown that permanent chemotherapy-induced alopecia in women with breast cancer, which is being increasingly documented [24,25], is associated with severe impairment of quality of life [26,27] and body image [28]. Persistent alopecia also has been associated with depressive symptoms, anxiety, and increased somatization among female survivors of childhood cancer [29]. Depressive and anxiety symptoms have similarly been reported among women living with alopecia as a result of a dermatological condition [19]. Furthermore, a significant negative impact has been found even among breast cancer patients with temporary [30,31] and mild alopecia [26,32]. 

Women with a childhood history of irradiation for tinea capitis represent another population warranting investigation for how persistent hair loss affects mental health. To our knowledge, only one study to date has examined this question [33], finding that compared to the general population of women in Israel, affected women report significantly higher rates of depression, antidepressant use, psychotherapy, psychiatric hospitalization, and attempted suicide. However, the association of hair loss severity with poorer mental health in this population is not known, and neither are the factors that contribute to these mental health outcomes. 

In the current study, we aimed to address this gap by examining the extent of the association between hair loss severity and poor mental health in women who had irradiation for tinea capitis during childhood and by identifying factors contributing to their mental health outcomes. 

## 2. Methods

Approval for a retrospective review of medical records, held at the archives of the Israel National Center for Compensation of Scalp Ringworm Victims, was obtained from the Human Subjects Research Committee of Ben-Gurion University of the Negev (BGU HSR 1427-1) and from the Helsinki Committee of Sheba Medical Center–Ramat Gan (SMC-2448-15 - Helsinki Sheba). We reviewed the medical records for all women who received compensation between 1995 and 2015 for full or partial alopecia resulting from irradiation treatment in childhood for tinea capitis. The compensation was given to these women under Article 77 of Israel National Insurance Ordinances that are related to alopecia universalis [34]. In our review, we identified 2509 medical records containing detailed information on women’s physical and mental health conditions. Data retrieval from these records was carried out in accordance with ethical standards for such work as set forth in Article 20 (A) (7) of Israel’s 1996 Patient’s Rights Law. The review and data retrieval took place between January 1, 2016, and December 31, 2017, and focused on demographic information, irradiation treatment (place, age), hair loss severity (defined using the disability allocation under national reimbursement criteria for alopecia: <15% vs 15–20%, the maximum compensation), mental health conditions (mood and anxiety disorders, emotional distress, low self-esteem, antidepressant use, suicidal ideation/attempts, psychotherapy) (yes/no), family and social problems (marital problems, parent–child relationship difficulties, bullying in childhood or adulthood, avoiding social settings because of hair loss) (yes/no), and physical health conditions including cancer, diabetes, hypertension, and migraines (yes/no). Information about mental and physical health conditions and social and family experiences was all derived from self-reports. 

### Statistical Analysis

To examine differences between women with high and low degrees of hair loss relative to other factors such as family status, unemployment, mental health conditions, and family and social problems, we performed chi-square tests for independence of measures with a Monte Carlo significance test followed by relative risk (RR) scores. To examine differences in age and years of education, we conducted independent-samples t-tests. 

Next, we conducted a hierarchical regression analysis to examine whether the degree of hair loss predicted the number of mental health problems that women experienced after controlling for background measures, family and social factors, health problems (migraines, diabetes, hypertension, and cancer), and age at radiation. In the first step of the analysis, we introduced background measures as predictors. In the second step, we added as predictors the family variables: intimate partner violence, child-to-parent violence, and parent–child relationship difficulties. In the third step of the analysis, we added social variables: social abuse during childhood, social abuse during adulthood, and avoidance of social situations. In the fourth step of the analysis, we added physical health variables: migraines, diabetes, hypertension, and cancer; and in the fifth step, we added age at radiation and level of hair loss (high = 1, low = 0). Additionally, we applied a multi-path mediation model using the MPlus 8.2 Structural Equation Modeling package [35] to examine whether family and/or social problems mediated the effect of hair loss severity on mental health (an additive index). In the model, hair loss severity (high = 1, low = 0) served as the predictor, and the outcome measure was mental health problems. Mediators were family (a latent factor loaded with the indices “intimate partner violence, parent–child relationship difficulties, and child to parent violence”) and/or social problems (a latent factor loaded with the indices “social abuse in childhood, social abuse in adulthood, and avoidance of social situations”). We also added as covariates age at the time of radiation and a latent factor loaded with the physical health problems (migraines, diabetes, hypertension, and cancer). Significance was estimated by bias-corrected bootstrap analysis with 1000 resampling cycles. 

Finally, we examined whether the rates of health problems among women with hair loss differed significantly from reported rates for the general population of women in Israel (ages 45–54 and 55–64 as reported in [36,37]) by employing a series of chi-square tests for goodness of fit and calculating RR scores.

## 3. Results

Among the 2509 women who received compensation for hair loss resulting from irradiation for tinea capitis, 1164 (46%) had high levels of hair loss and 1345 (54%) had low levels of hair loss. Power analyses indicated that the sample size allowed for 80% power in detecting effect sizes as small and subtle as an odds ratio of 1.15 (used in logistic regressions), ω = 0.06 (used in chi-square–based tests), and Cohen’s f2 = 0.003 (used in linear regressions). Overall, 2.0% of the data were missing, and Little’s missing completely at random test indicated that they were missing completely at random (χ^2^(2) = 0.02, *p* = 0.99). Accordingly, missing data were handled by multiple imputation with 10 impute databases [38]. All analyses were based on the pooled multiple imputation results. Table 1 presents the characteristics of the study sample (N = 2509).

### 3.1. Background Measures

Women with more hair loss were younger (M = 56.32, SD = 7.62, t = 2.83, *p* = 0.005, 95% CI Hedges’s *g* = −0.19 to −0.11) and had less formal education (M = 8.70, SD = 3.31, t = 3.89, *p* < 0.001, 95% CI Hedges’s *g* = −0.29 to −0.19) than women with lower levels of hair loss (age: M = 57.17, SD = 7.23; education: M = 9.34, SD = 3.42). In addition, the prevalence of marriage was lower among women with higher levels of hair loss (64.3% [743]) compared with that of women with lower levels of hair loss (71.6% [963]; χ^2^(1) = 15.49, *p* < 0.001, φ = 0.08). We found no differences in percentages of unemployment (23.0% with high hair loss levels and 22.2% for women with low hair loss; χ^2^(1) = 0.20, *p* = 0.67, φ = 0.01).

### 3.2. Mental Health Conditions

As shown in Table 2, compared with women with low levels of hair loss, significantly higher proportions of women with high levels of hair loss reported depression symptoms, emotional distress, social anxiety, low self-esteem, and suicidal ideation. Conversely, fewer women with high hair loss levels reported using antidepressants compared with women with low levels of hair loss, and a similar number of women in both groups received psychotherapy. Women with high levels of hair loss had an especially high risk for self-reported social anxiety (RR, 2.44; 95% CI, 2.09–2.87; *p* < 0.005).

### 3.3. Family and Social Problems

As shown in Table 3, compared with women who had low levels of hair loss, women with high levels of hair loss were significantly more likely to report marital problems, intimate partner violence, social abuse during childhood and adulthood, and avoidance of social situations because of hair loss. They had the highest risk for self-reported social abuse during adulthood (RR, 2.46; 95% CI, 1.66–3.54; *p* < 0.005) and social avoidance (RR, 1.76; 95% CI, 1.53–2.02; *p* < 0.005).

### 3.4. Predictors of Mental Health Outcomes

Hierarchical regression coefficients are presented in Table 4. The analysis indicated that the background measures explained 2% of the variance, with older and unmarried women experiencing more mental health problems than younger and married women. The family variables added 9.7% of the variance, with intimate partner violence and parent–child relationship difficulties being associated with more mental health problems. The social variables added 15.9% to the explained variance, with abuse during childhood and social avoidance because of hair loss linked to more mental health problems. Including the physical health problems in the analysis added only 1.2% to the explained variance of mental health problems, with weak effects: women with migraines, hypertension, and/or cancer had more mental health problems than those who were not exposed to these conditions. Finally, adding the hair loss measures added 2.1% to the explained variance, with high levels of hair loss being related to more mental health problems beyond the contribution of background characteristics, family factors, and social and physical health problems. Age at radiation also significantly predicted the number of mental health problems, with a younger age at radiation being associated with more mental health problems. Overall, the model explained 31.0% of the variance in women’s mental health.

### 3.5. Test of Mediation

**Figure 1:**
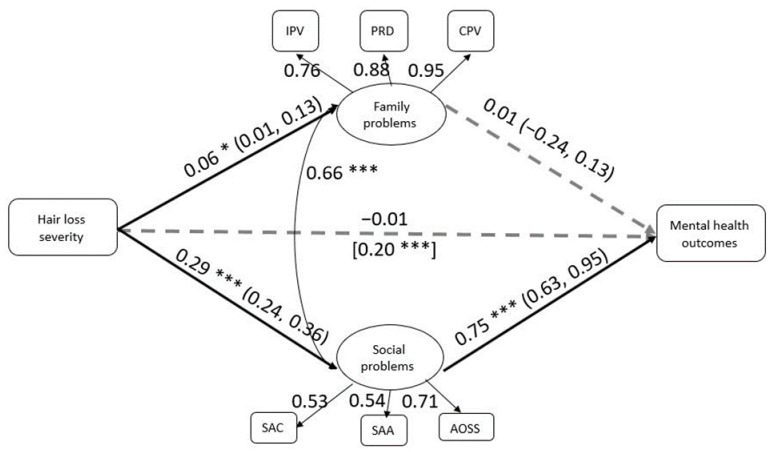
The mediation model. Note. IPV = intimate partner violence; PRD = parent–child relationship difficulties; CPV = child-to-parent violence; SAC = social abuse during childhood; SAA = social abuse during adulthood; AOSS = avoidance of social situations. * *p* < 0.05, *** *p* < 0.001. Solid lines mark significant paths, and dashed lines mark nonsignificant paths. Value in brackets refer to the total effect of hair loss on mental health (i.e., before the mediation). Values in parentheses refer to 95% confidence intervals for the mediation paths.

We examined whether women’s family and/or social problems mediated the effect of hair loss severity on mental health problems. The model is presented in Figure 1. The model had adequate fit to the observed data (*CFI* = 0.95, *TLI* = 0.92, *RMSEA* = 0.04). The results indicate that women’s social problems significantly and fully mediated the effect of hair loss severity on mental health problems (indirect = 0.72; 95% bias-corrected confidence interval, 0.53–1.08). Specifically, women with more severe hair loss reported more social problems (i.e., experiencing social abuse in childhood and/or adulthood and avoiding social situations), and having more social problems was related to reporting more mental health problems. This mediation path was significant, controlling for the effect of physical health problems and age at radiation. Women’s family problems did not significantly mediate the effect of hair loss on mental problems.

### 3.6. Physical Health Conditions

The percentage of all included physical health conditions was significantly higher among women with hair loss compared with women aged 45–54 in the general population. They had an especially high risk of migraines (RR, 5.25; 95% CI, 4.45–6.20; *p* < 0.001) and cancer (RR, 17.90; 95% CI, 9.96–32.17; *p* < 0.001). Women with hair loss were also at greatest risk for migraines and cancer compared with women aged 55–64 (migraines: RR, 6.29; 95% CI, 5.10–7.75; *p* < 0.001; cancer: 4.66; 95% CI, 3.44–6.32; *p* < 0.001) and women aged 65+ (migraines: RR, 6.80; 95% CI, 5.33–9.14; *p* < 0.001; cancer: RR, 3.01; 95% CI, 2.42–3.76; *p* < 0.001) in the general population (see Table S1).

## 4. Discussion

This study focused on mental health outcomes among women irradiated for tinea capitis as children, one of the few groups to experience the long-term impacts of persistent alopecia from childhood. Our results indicate that women with higher levels of hair loss have an increased risk of reporting a range of mental health problems, especially social anxiety. These results suggest that women with higher degrees of hair loss were more psychologically affected compared with their counterparts with lower degrees of loss. Severe hair loss could have greater negative effects across various domains of women’s lives that hair loss may affect, including sense of femininity, sexuality, and attractiveness, self-perception of appearance, body image, self-esteem, and social functioning [28,39,40,41,42]. The high prevalence of social anxiety among women with severe hair loss we identified here reinforces previous findings indicating clinically significant levels of social anxiety among women with different types of alopecia [19]. Patients with alopecia often develop a secondary social anxiety disorder that manifests in anxiety symptoms similar to those of the primary type [43].

Previous findings indicate that a substantial proportion of women with alopecia experience marital problems [44], possibly related to the negative impact of hair loss on sexual quality of life [45]. Our findings extend these previous results, linking a higher degree of hair loss in women with increased risk for experiencing marital problems, including physical abuse. The higher rates of divorced and never-married women among those with more severe hair loss is further evidence that hair loss severity can affect partner relationships.

We also found that women with higher degrees of hair loss were more likely to report having experienced social abuse, particularly during their adult years, resulting in an increased tendency to avoid social situations. By linking women’s social experiences to the severity of their hair loss, these findings add to earlier reports documenting the devastating social consequences of different types of alopecia for women [41,44,46,47].

The results of our regression analyses reveal that hair loss severity is a significant predictor of women’s mental health, distinct from other predictors such as background characteristics, family issues, and social and physical health problems. Prior research has suggested that hair loss severity does not necessarily predict a woman’s quality of life [48]. The reason for this contrasting finding may be traced to the use of the Skindex-16 instrument, which measures the extent to which patients’ psychosocial experiences bother them, rather than the rates of their psychosocial experiences [49] that we measured here.

Family and social problems emerged as the strongest predictors of self-reported mental health outcomes of the women in this study. One of our aims was to examine whether these problems mediate the effect of hair loss severity on mental health. Our path analysis revealed that hair loss severity has an indirect effect on mental health through an impact on social life. These results highlight the prominent role in both childhood and adulthood of social experiences related to hair loss in shaping mental health outcomes among women. This finding also supports previous evidence linking childhood appearance-related teasing experiences with depression, anxiety, and low self-esteem in women [50]. Of interest, we found that women who were irradiated at a younger age experienced more mental health problems. One possible explanation for this finding is that a longer exposure to appearance-related teasing has a greater negative impact on mental health outcomes, suggesting an incremental dose effect.

Our study shows that compared with women in the general population of Israel, women with alopecia resulting from irradiation treatment for tinea capitis are at increased risk for serious physical conditions, with an especially high risk of developing migraines and cancer. Cancer prevalence has already been strongly associated with a history of childhood irradiation for tinea capitis [6,9,10,11,12,13]. Although studies have not specifically addressed the association of this childhood exposure with migraines, migraines are associated with early childhood trauma and adult stressful life events [51,52]. 

A major limitation of this study is its retrospective nature. The primary disadvantages of retrospective review of medical records include missing data within the medical record, difficulty in interpreting documented information, and variability in terms of the quality of the documented information [53]. A second limitation is that the data regarding women’s mental health conditions are primarily self-reported, without confirmation of clinical diagnoses, and associated with applications for medical compensation. Given the nature of these applications, some magnification of negative experiences is possible. The reliance on self-reported data, however, also reflects a major strength of the current study, because this information reveals the subjective perspectives and experiences of women living with alopecia resulting from irradiation for tinea capitis in childhood. A third limitation to consider is that the women in this study may have coped with additional forms of alopecia besides post irradiation-induced hair loss, such as androgenetic alopecia, alopecia areata, and cicatricial, which were not mentioned in their medical records. It is, therefore, possible that additional forms of alopecia that coexist with alopecia resulting from irradiation treatment for tinea capitis also account for the variance in the mental health condition of these women.

## 5. Conclusions

Our results indicate that hair loss severity in women irradiated for tinea capitis in childhood is associated with an increased risk for later self-reported mental health problems. Negative social experiences mediate these effects of hair loss severity on mental health outcomes. These results suggest that healthcare professionals supporting women with hair loss after irradiation for childhood tinea capitis should be alert to a history of severe levels of hair loss, given the risk for important mental health consequences. Our results also stress the need for health policy makers to develop comprehensive services designed to meet the needs of these women, based on a whole-person care approach that takes into account the physical, emotional and social aspects of a patient’s health, and addresses them in an integrated format [54,55]. By doing so, they may improve the well-being of these women.

The contribution of the study goes beyond the specific case of hair loss resulting from childhood irradiation for tinea capitis, as it provides insight into the pathway through which hair loss severity can influence the mental health of affected women. Further research is needed to investigate the impacts on women’s mental health of hair loss severity associated with other diseases.

## Figures and Tables

**Table 1:** Study Participant Characteristics.

Characteristics		%(n) or Mean (SD)
Age		56.79 (±7.43)
Country of birth	Israel	16.3 (300)
	Europe	1.9 (34)
	Asia/Africa	81.4 (1489)
	America	0.4 (7)
Employed		77.4 (1522)
Family status	Married	70.3 (1711)
	Divorced	12.9 (312)
	Widower	12 (292)
	Single	4.9 (119)
Years of education		9.05 (±3.39)
Place of radiation	Israel	55.9 (1386)
Radiation age	0–5 years	28.3 (711)
	6–10 years	50.6 (1271)
	11–15 years	21 (527)
High percentage (15%–20%) of disability		46.4 (1164)
under Article 77		
Use of wig		45.2 (1105)
Suicide attempts		1.1 (27)
Depression		45.5 (1129)
Emotional distress		60.1 (1489)
Anxiety		35.7 (886)
Low self-esteem		56.6 (1409)
Social anxiety		22.6 (562)
Suicidal ideation		5.3 (132)
Psychotherapy		22.5 (553)
Use of antidepressants		25.7 (630)
Marital problems		22.6 (546)
Intimate partner violence		22.6 (546)
Parent–child relationship difficulties		6.2 (151)
Child-to-parent violence		1.3 (31)
Social abuse during childhood		31.8 (789)
Social abuse during adulthood		4.7 (117)
Avoidance of social situations		25.2 (621)
Migraines		60.4 (1501)
Diabetes		17.8 (436)
Hypertension		22.3 (541)
Cancer		40.8 (1006)

Note. N = 2509. Values for age and years of education are means and standard deviations (in parentheses). Other values are rates and frequencies (in parentheses).

**Table 2:** Mental health conditions.

Variables	Hair loss				*χ* ^2^	RR (95% CI)
	Low		High			
	%	*n*	%	*n*		
Suicide attempts	1.2	16	1	11	0.34	0.80 (0.37, 1.71)
Depression	39.9	534	51.7	595	34.48 ***	1.29 (1.19, 1.41)
Emotional distress	52.7	702	68.5	787	64.11 ***	1.30 (1.21, 1.39)
Anxiety	35.6	474	36	412	0.06	1.01 (0.91, 1.13)
Low self-esteem	47.2	633	67.5	776	103.20 ***	1.43 (1.33, 1.53)
Social anxiety	13.6	180	33.2	382	135.59 ***	2.44 (2.09, 2.87)
Suicidal ideation	4.4	59	6.3	73	4.58 *	1.43 (1.03, 2.01)
Psychotherapy	22.3	296	22.6	257	0.04	1.01 (0.88, 1.18)
Antidepressants/anti-anxiety drugs	29	379	22.1	251	15.32 ***	0.76 (0.66, 0.87)

Note. RR = relative risk; 95% CI = 95% confidence interval. * *p* < 0.05, *** *p* < 0.005.

**Table 3:** Family and social problems.

Variables	Hair loss				*χ* ^2^	RR (95% CI)
	Low		High			
	%	*n*	%	*n*		
Marital problems	20.1	264	25.3	282	9.23 *	1.26 (1.08, 1.45)
Intimate partner violence	20.1	264	25.3	282	9.23 *	1.26 (1.08, 1.45)
Parent–child relationship difficulties	6	78	6.5	73	0.29	1.08 (0.80, 1.48)
Child-to-parent violence	1.4	18	1.2	13	0.26	0.89 (0.40, 1.69)
Social abuse during childhood	27.5	366	36.8	423	24.70 ***	1.34 (1.19, 1.50)
Social abuse during adulthood	2.8	38	6.9	79	22.58 ***	2.46 (1.66, 3.54)
Avoidance of social situations	18.7	248	32.9	373	65.73 ***	1.76 (1.53, 2.02)

Note. RR = relative risk; 95% CI = 95% confidence interval. * *p* < 0.05, *** *p* < 0.005.

**Table 4:** Regression coefficients for predicting women’s mental health.

Steps	Predictors	*b* (95% CI)	*β*
Step 1	Age	0.02 (0.01, 0.03)	0.10 ***
	Years of education	0.003 (−0.02, 0.03)	0.01
	Family status (1 = married)	−0.33 (−0.51, −0.15)	−0.10 ***
	*R* ^2^	2.00%	
Step 2	Age	0.03 (0.02, 0.04)	0.12 ***
	Years of education	0.01 (−0.01, 0.03)	0.02
	Family status (1 = married)	−0.24 (−0.42, −0.07)	−0.07 **
	Intimate partner violence	1.00 (0.81, 1.20)	0.27 ***
	Parent-child relationship difficulties	0.67 (0.31, 1.03)	0.11 ***
	Child-to-parent violence	−0.15 (−0.88, 0.58)	−0.01
	*ΔR* ^2^	9.70%	
Step 3	Age	0.03 (0.02, 0.04)	0.12 ***
	Years of education	0.01 (−0.01, 0.04)	0.03
	Family status (1 = married)	−0.20 (−0.35, −0.04)	−0.06 *
	Intimate partner violence	0.61 (0.42, 0.79)	0.16 ***
	Parent-child relationship difficulties	0.52 (0.19, 0.84)	0.08 **
	Child-to-parent violence	−0.33 (−0.99, 0.34)	−0.02
	Abuse during childhood	0.68 (0.52, 0.83)	0.20 ***
	Abuse during adulthood	0.18 (−0.18, 0.55)	0.02
	Avoidance of social situations	1.19 (1.02, 1.35)	0.33 ***
	*ΔR* ^2^	15.90%	
Step 4	Age	0.03 (0.02, 0.04)	0.12 ***
	Years of education	0.01 (−0.01, 0.04)	0.03
	Family status (1 = married)	−0.20 (−0.36, −0.04)	−0.06 ***
	Intimate partner violence	0.61 (0.43, 0.79)	0.16 ***
	Parent-child relationship difficulties	0.45 (0.13, 0.77)	0.07 *
	Child-to-parent violence	−0.24 (−0.90, 0.42)	−0.02
	Abuse during childhood	0.64 (0.49, 0.80)	0.19 ***
	Abuse during adulthood	0.21 (−0.15, 0.57)	0.03
	Avoidance of social situations	1.17 (1.00, 1.35)	0.32 ***
	Migraines	0.25 (0.10, 0.39)	0.08 **
	Diabetes	0.03 (−0.17, 0.24)	0.01
	Hypertension	0.20 (0.01, 0.39)	0.05 *
	Cancer	0.16 (0.02, 0.31)	0.05 *
	*ΔR* ^2^	1.20%	
Step 5	Age	0.04 (0.03, 0.05)	0.18 ***
	Years of education	0.01 (−0.01, 0.03)	0.02
	Family status (1 = married)	−0.15 (−0.30, 0.01)	−0.04
	Partner violence	0.59 (0.41, 0.77)	0.16 ***
	Parent-child relationship difficulties	0.47 (0.15, 0.79)	0.07 *
	Child-to-parent violence	−0.17 (−0.82, 0.45)	−0.01
	Abuse during childhood	0.63 (0.48, 0.78)	0.19 ***
	Abuse during adulthood	0.14 (−0.22, 0.50)	0.02
	Avoidance of social situations	1.13 (0.96, 1.29)	0.31 ***
	Migraines	0.27 (0.13, 0.42)	0.08
	Diabetes	0.03 (−0.17, 0.23)	0.01
	Hypertension	0.23 (0.04, 0.41)	0.06 *
	Cancer	0.18 (0.04, 0.32)	0.06 *
	Age at radiation	−0.24 (−0.36, −0.12)	−0.10 ***
	Hair loss	0.42 (0.27, 0.56)	0.13 ***
	*ΔR* ^2^	2.10%	
	Overall *R*^2^	cc	

Note. 95% CI = 95% confidence interval. * *p* < 0.05, ** *p* < 0.01, *** *p* < 0.001.

## Supplementary Materials

The following are available online at https://www.mdpi.com/1660-4601/17/20/7388/s1, Table S1. Rates of health problems among women with hair loss compared to women in the general population in Israel.
