# Supplementary material for: Does Severity of Hair Loss Matter? Factors Associated with Mental Health Outcomes in Women Irradiated for Tinea Capitis in Childhood
Source: Int J Environ Res Public Health. 2020 Oct 10;17(20):7388. doi: 10.3390/ijerph17207388 (PMC7601621; doi:10.3390/ijerph17207388)
Supplement: Supplementary file 1 [file ijerph-17-07388-s001.pdf]

International Journal of
Environmental Research and Public Health

MDPI

Table 1. Rates of health problems among women with hair loss compared to women in the general population in Israel.

		General population: ages 45-54			General population: ages 55-64		
	Hair loss	% (n)	χ^2	***RR* (95% CI)**	**% (n)**	χ^2	***RR* (95% CI)**
Migraines	60.4% (1501)	11.5% (128) [1]	5847.20***	5.25 (4.45 6.20)	9.6% (81)	7400.73***	6.29 (5.10 7.75)
Diabetes	17.8% (436)	8.1% (39) [2]	311. 45***	2.21 (1.61 3.01)	14.2% (63)	26.57***	1.26 (0.98 1.61)
Hypertension	22.3% (541)	15.8% (76) [2]	91.34***	1.42 (1.14 1.76)	31.2% (139)	89.14***	0.72 (0.61 0.84)
Cancer	40.8% (1006)	2.2% (11) [2]	7172.85***	17.90 (9.96 32.17)	8.8% (39)	3253.90***	4.66 (3.44 6.32)

	General population: ages 65+		
	% (n)	χ^2	***RR* (95% CI)**
Migraines	8.6% (49) [1]	8488.18***	6.80 (5.33 9.14)
Diabetes	20.5% (109) [2]	10.61*	0.87 (0.72 1.05)
Hypertension	52.6% (280) [2]	888.78***	0.42 (0.38 0.47)
Cancer	13.6% (72) [2]	1557.12***	3.01 (2.42 3.76)

Note. . * $p < .05$, *** $P < .001$. RR = relative risk; 95% CI = 95% confidence interval. Superscript numbers [1, 2] represent the references in the References list from which the data on the general population of women in Israel was extracted: [1] Israeli National Health Interview Survey (INHIS-1), 2003-2004: selected findings. (2006). Israel Center for Disease Control, Ministry of Health; [2] Israel National Health Interview Survey (INHIS-3), 2013–2015: selected findings. (2017). Israel Center for Disease Control, Ministry of Health.

© 2020 by the authors. Licensee MDPI, Basel, Switzerland. This article is an open access article distributed under the terms and conditions of the Creative Commons Attribution (CC BY) license (http://creativecommons.org/licenses/by/4.0/).
